# Lack of HIV RNA test result is a barrier to breastfeeding among women living with HIV in Botswana

**DOI:** 10.1186/s13006-021-00424-x

**Published:** 2021-10-13

**Authors:** Gloria Katuta Mayondi, Aamirah Mussa, Rebecca Zash, Sikhulile Moyo, Arielle Issacson, Modiegi Diseko, Judith Mabuta, Goabaone Mogomotsi, Eldah Dintwa, Joseph Makhema, Mompati Mmalane, Shahin Lockman, Chelsea Morroni, Roger Shapiro

**Affiliations:** 1 Botswana-Harvard AIDS Institute Partnership, Gaborone, Botswana; 2 Department of Immunology and Infectious Diseases, Harvard T.H. Chan School of Public Health, Boston, MA USA; 3 Division of Infectious Diseases, Beth Israel Deaconess Medical Center, Boston, MA USA; 4 Harvard Medical School, Boston, MA USA; 5 Ministry of Health and Wellness, Gaborone, Botswana; 6 Division of Infectious Disease, Brigham and Women’s Hospital, Boston, MA USA; 7 MRC Centre for Reproductive Health, University of Edinburgh, Edinburgh, UK

**Keywords:** HIV, Infant feeding, Women living with HIV, Breastfeeding, Viral load, Botswana

## Abstract

**Background:**

Botswana updated its antiretroviral treatment (ART) guidelines in May 2016 to support breastfeeding for women living with HIV (WLHIV) on ART who have documented HIV RNA suppression during pregnancy.

**Methods:**

From September 2016 to March 2019, we evaluated feeding method at discharge among WLHIV at eight government maternity wards in Botswana within the Tsepamo Study. We validated the recorded feeding method on the obstetric record using the prevention of mother-to-child transmission of HIV (PMTCT) counsellor report, infant formula dispensing log or through direct observation. Available HIV RNA results were recorded from the obstetric record, and from outpatient HIV records (starting February 2018). In a subset of participants, we used electronic laboratory records to verify whether an HIV RNA test had occurred. Univariable and multivariable logistic regression analyses were performed to identify factors associated with infant feeding choice.

**Results:**

Among 13,354 WLHIV who had a validated feeding method at discharge, 5303 (39.7%) chose to breastfeed and 8051 (60.3%) chose to formula feed. Women who had a documented HIV RNA result in the obstetric record available to healthcare providers at delivery were more likely to breastfeed (50.8%) compared to women who did not have a documented HIV RNA result (35.4%) (aOR 0.59; 95% CI 0.54, 0.65). Among women with documented HIV RNA, 2711 (94.6%) were virally suppressed (< 400 copies/mL). Breastfeeding occurred in a substantial proportion of women who did not meet criteria, including 46 (30.1%) of 153 women with HIV RNA > 400 copies/mL, and 134 (27.4%) of 489 women with no reported ART use. A sub-analysis of electronic laboratory records among 150 women without a recorded result on the obstetric record revealed that 93 (62%) women had an HIV RNA test during pregnancy.

**Conclusions:**

In a setting of long-standing use of suppressive ART, with majority of WLHIV on ART from the time of conception, requiring documentation of HIV RNA suppression in the obstetric record to inform infant feeding decisions is a barrier to breastfeeding but unlikely to prevent a substantial amount of HIV transmission.

## Background

Feeding method in the first 6 months of life plays an integral role in a child’s health and development trajectory [1]. Breastfeeding is one of the foundations of child health and development and is associated with substantially lower child morbidity and mortality, including among HIV-exposed children [2, 3]. The 2010 and 2016 World Health Organization (WHO) guidelines on HIV and infant feeding define a primary goal of maximizing infant HIV-free survival [2–4] and support breastfeeding in the setting of maternal antiretroviral therapy (ART) or infant antiviral prophylaxis. In low-resource settings where diarrhoeal diseases, pneumonia and malnutrition are common causes of infant morbidity and mortality, WHO recommends exclusive breastfeeding for 6 months and continued breastfeeding up to 24 months with maternal or infant ARV protection in order to give infants the best chance for HIV-free survival [2, 5].

Despite outstanding ART coverage in pregnancy (95%), with 64.5% of WLWHIV conceiving on ART and very low (< 1.5%) mother-to-child HIV transmission (MTCT) [6, 7], Botswana has been slow to adopt a policy of universal breastfeeding for HIV-exposed infants. With over 20% of infants in the country HIV-exposed [8, 9] the lack of breastfeeding for these children contributes substantially to Botswana’s high under-five mortality (41.6 deaths per 1000 live births) compared to the Sustainable Development Goal target to reduce under-five mortality to at least as low as 25 deaths per 1000 live births [10, 11]. In recognition of this problem, Botswana chose to align its guidelines with those of the WHO in May 2016 to encourage greater breastfeeding for women living with HIV (WLHIV) on ART [12]. However, because Botswana can perform HIV RNA (viral load) testing as part of its ART program, the 2016 Botswana guidelines also specified that there should be documented HIV RNA suppression within the last 3 months of pregnancy to support a recommendation to breastfeed at the time of delivery. The updated guidelines also retain an individualised approach to feeding, recommending counselling during pregnancy to ensure each woman makes the best decision for her situation.

After decades of an MTCT prevention programme that relied on the use of formula, and with the additional requirement of documented viral suppression in the third trimester of pregnancy to support breastfeeding, it is currently unknown how the change in 2016 guidelines impacted actual feeding choices in Botswana. Therefore, we set out to describe feeding choices and potential barriers to breastfeeding among WLHIV in Botswana after the new guidelines were implemented.

## Methods

### Study design and setting

#### Tsepamo study overview

**Fig. 1:**
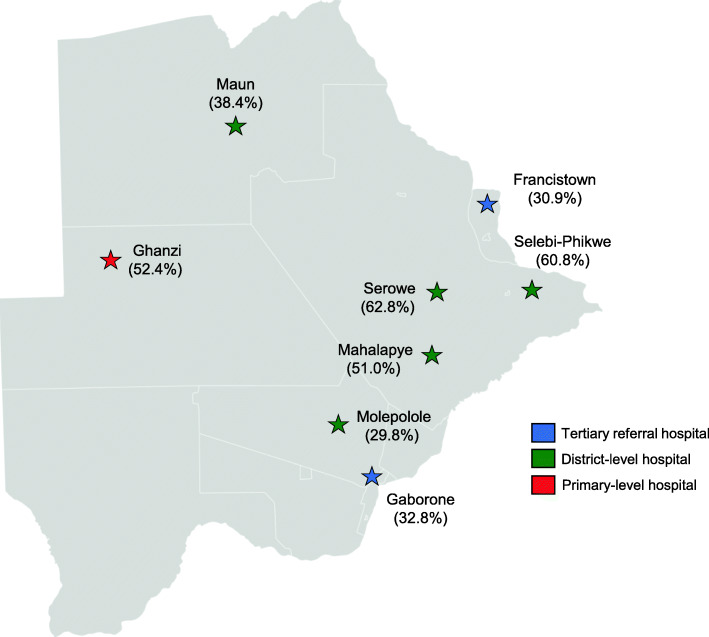
Tsepamo study sites and percentage of WLHIV breastfeeding at discharge at each site

We performed an analysis of infant feeding and HIV RNA data collected in the Tsepamo Study, an ongoing non-interventional birth outcomes surveillance study that collects data from obstetric records at large public maternity wards throughout Botswana [13]. The primary study aims of Tsepamo are to evaluate adverse birth outcomes and congenital abnormalities by HIV status and ART regimen. The study occurs at geographically distributed sites in major population areas of Botswana (Fig. 1), where > 95% of women deliver in a healthcare facility. Although the study expanded from eight to 18 sites in 2018, only data from the original eight sites were included in this analysis; these sites were located in Gaborone and Francistown (tertiary referral centres) and Maun, Serowe, Selebi-Phikwe, Mahalapye, Molepolole and Ghanzi (district and primary-level hospitals).

### Data collection

Data were abstracted from obstetric record cards into an electronic database by trained research assistants at the time of maternal discharge from the postnatal ward. The obstetric record card is a standardized government booklet to record the entirety of medical care during pregnancy and delivery, that is started at the first antenatal clinic (ANC) visit, and brought by the mother to each subsequent ANC visit and to the delivery site. Information extracted from the obstetric record included maternal demographic characteristics, medical history, medications prescribed at the time of conception and during pregnancy, maternal diagnoses during pregnancy, infant birth record, type of delivery, APGAR scores, gestational age, birthweight, congenital abnormalities, and vital status of the infant(s) at time of discharge. For WLHIV, the date of HIV diagnosis, most recent CD4 cell count, and antiretroviral history (including start date, regimen, and any switch or discontinuation during pregnancy) were also extracted. When available in the obstetric record, HIV RNA results were recorded. Births that occurred before arrival at the hospital and < 24 weeks gestation were excluded.

For this analysis, we used data collected between September 2016 and March 2019 and women were included if they were living with HIV, if their baby was alive at the time of discharge, and if they had a validated feeding method recorded at discharge. Validation started in September 2016 and occurred through direct feeding observation or by checking the prevention of mother-to-child transmission of HIV (PMTCT) counsellor report or formula dispensing log.

All HIV RNA results documented in the obstetric record were abstracted. We verified a subset of randomly selected records from our sample (150 with and 150 without a documented HIV RNA result in the obstetric record) to determine whether an HIV RNA test had occurred during pregnancy. This verification was performed using the Integrated Patient Management System (IPMS), a nationwide electronic system that includes laboratory records. After February 2018, we also evaluated participants’ Infectious Disease Control Centre (IDCC) cards (the medical record for outpatient HIV care), when available, to further identify HIV RNA results missing from the available obstetric record. However, it should be noted that the obstetric record was the only source of information routinely available through the Botswana PMTCT programme to help midwives in counselling for appropriate feeding recommendations at the time of delivery. While the IPMS captures laboratory records nationwide, not all maternity facilities have access to these electronic laboratory records. Laboratory results of participants are routinely sent non-electronically to antenatal facilities for the nursing staff to transcribe them into participants’ obstetric records.

### Statistical analysis

Data were extracted from the electronic database in an excel format and analysed in Stata (Version 16, StataCorp, College Station, Texas). Descriptive statistics were used to describe the infant feeding choices of WLHIV (proportions of women in each feeding group). To identify factors associated with infant feeding choices of WLHIV, we performed univariable and multivariable logistic regression analyses. Infant feeding choice was categorized as breastfeeding versus formula feeding where breastfeeding was used as the reference category in logistic regression models. All independent variables that were significant or nearly significant in univariable analysis (*P* < 0.1) were included in the multivariable model. Statistical significance was inferred at a *P*-value of < 0.05. The outcome variable used was feeding choice at discharge. Independent variables of interest included age, marital status, education, occupation, nationality, delivery site, received antenatal care, documented viral load during pregnancy on the obstetric record, ART status during pregnancy, and gravida.

## Results

### Population and characteristics of the study sample

**Fig. 2:**
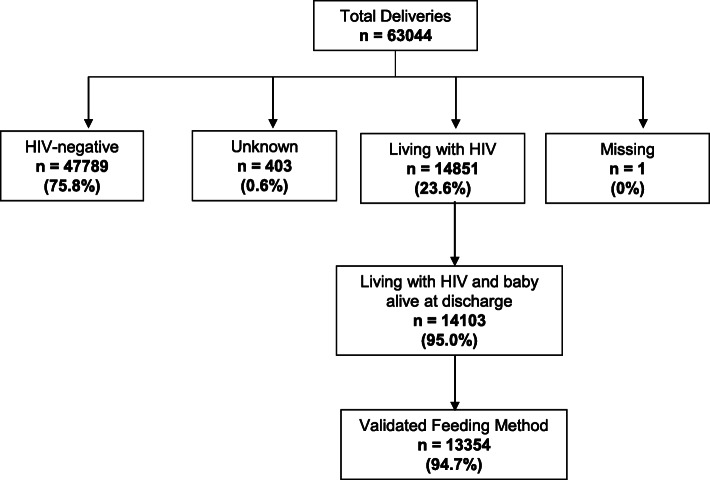
Study population diagram

There were 63,044 total deliveries at the eight maternity sites between September 2016 and March 2019, with 14,851 (23.6%) infants born to WLHIV. Of these WLHIV, 14,103 (95.0%) had a baby that was discharged alive and 13,354 (94.7%) of them had a validated feeding method recorded (Fig. 2). ART coverage was high, with 96.3% of women in the study receiving ART during pregnancy: 8598 (64.8%) started ART before conception and 4175 (31.5%) initiated ART during pregnancy. Of those with a known ART start date, 12,200 (96.6%) had received at least 8 weeks of ART before delivery. Table 1 shows the baseline characteristics of the study sample.

**Table 1:** Characteristics of the study sample, by feeding status at discharge

	Total ***N*** (%)	Breastfeeding at discharge ***n*** (%)	Formula feeding at discharge ***n*** (%)
**Age**			
Under 18	122 (0.9)	44 (36.1)	78 (63.9)
18–29	5460 (40.9)	2262 (41.4)	3198 (58.6)
30–39	6722 (50.4)	2623 (39.0)	4099 (61.0)
40 and older	1046 (7.8)	374 (35.8)	672 (64.2)
**Marital Status**			
Single	11,682 (89.9)	4624 (39.6)	7058 (60.4)
Married	1243 (9.6)	508 (40.9)	735 (59.1)
Widowed/divorced	75 (0.6)	24 (32.0)	51 (68.0)
**Education**			
Primary and below	1539 (11.7)	622 (40.4)	917 (59.6)
Secondary	9957 (75.7)	3894 (39.1)	6063 (60.9)
Tertiary	1653 (12.6)	710 (43.0)	943 (57.1)
**Occupation**			
Unemployed/student	8133 (63.2)	3275 (40.3)	4858 (59.7)
Salaried employment	4739 (36.8)	1834 (38.7)	2905 (61.3)
**Nationality**			
Motswana	12,972 (97.5)	5137 (39.6)	7835 (60.4)
Other	337 (2.5)	156 (46.3)	181 (53.7)
**Delivery Site**			
Primary/district hospitals	6892 (51.6)	3247 (47.1)	3645 (52.9)
Tertiary hospitals	6462 (48.4)	2056 (31.8)	4406 (68.2)
**Attended antenatal visits**			
No	383 (2.9)	102 (26.6)	281 (73.4)
Yes	12,943 (97.1)	5188 (40.1)	7755 (59.9)
**Documentation of viral load during pregnancy**			
Yes	2865 (25.7)	1456 (50.8)	1409 (49.2)
No	8296 (74.3)	2936 (35.4)	5360 (64.6)
**ART status during pregnancy**			
No documentation of ART in pregnancy	489 (3.7)	134 (27.4)	355 (72.6)
ART initiated prior to pregnancy	8598 (64.8)	3360 (39.1)	5238 (60.9)
ART initiated during pregnancy	4175 (31.5)	1778 (42.6)	2397 (57.4)
**Gravida**			
Primigravida	1953 (14.7)	840 (43.0)	1113 (57.0)
Multigravida	11,370 (85.3)	4454 (39.2)	6916 (60.8)

### Feeding choice and associated factors

At discharge, 5303 (39.7%) of the WLHIV with a validated feeding method were confirmed to be breastfeeding and 8051 (60.3%) were formula feeding. The percentage of women breastfeeding increased by only 1.3% between 2016/2017 to 2018/2019.

In multivariable analysis, age, education, nationality, delivery site, attended antenatal visits, documentation of viral load during pregnancy and ART status during pregnancy (i.e., whether there was documentation of ART during pregnancy and whether ART was initiated prior to or during pregnancy) were associated with feeding choice (Table 2). Women aged 40 and older were the least likely to breastfeed, whereas those with a tertiary level of education or non-citizens of Botswana were more likely to breastfeed. Women who did not attend antenatal visits before delivery were less likely to breastfeed, as were women who delivered in a tertiary hospital (urban setting) rather than a primary/district hospital (rural or peri-urban setting). Women with no documentation of ART during pregnancy were less likely to be breastfeeding at discharge compared with women who either initiated ART before pregnancy or during pregnancy.

**Table 2:** Univariable and multivariable analysis of characteristics associated with lack of breastfeeding among WLHIV in the Tsepamo Study

	Total	OR (95% CI)	***P***-value	aOR (95% CI)	***P***-value
**Age**	**13,350**				
Under 18	122	1.13 (0.78, 1.65)	0.507	1.02 (0.64, 1.63)	0.942
18–29	5460	0.90 (0.84, 0.97)	0.007	0.94 (0.86, 1.04)	0.224
30–39	6722	Ref	Ref	Ref	Ref
40 and older	1046	1.15 (1.00, 1.32)	0.044	1.17 (1.00, 1.35)	0.043
**Marital Status**	**13,000**				
Single	11,682	Ref	Ref	–	–
Married	1243	0.95 (0.84, 1.07)	0.378	–	–
Widowed/divorced	75	1.39 (0.86, 2.26)	0.183	–	–
**Education**	**13,149**				
Primary and below	1539	0.95 (0.85, 1.06)	0.328	0.98 (0.86, 1.11)	0.739
Secondary	9957	Ref	Ref	Ref	Ref
Tertiary	1653	0.85 (0.77, 0.95)	0.003	0.83 (0.73, 0.94)	0.004
**Occupation**	**12,872**				
Unemployed/student	8133	Ref	Ref	Ref	Ref
Salaried employment	4739	1.07 (0.99, 1.15)	0.08	0.98 (0.90, 1.07)	0.672
**Nationality**	**13,309**				
Motswana	12,972	Ref	Ref	Ref	Ref
Other	337	0.76 (0.61, 0.95)	0.014	0.43 (0.33, 0.57)	< 0.001
**Delivery Site**	**13,354**				
Primary/district hospitals	6892	Ref	Ref	Ref	Ref
Tertiary hospitals	6462	1.91 (1.78, 2.05)	< 0.001	1.92 (1.77, 2.08)	< 0.001
**Attended antenatal visits**	**13,326**				
No	383	1.84 (1.47, 2.32)	< 0.001	1.64 (1.20, 2.23)	0.002
Yes	12,943	Ref	Ref	Ref	Ref
**Documentation of viral load during pregnancy**	**11,161**				
Yes	2865	0.53 (0.49, 0.58)	< 0.001	0.59 (0.54, 0.65)	< 0.001
No	8296	Ref	Ref	Ref	Ref
**ART status during pregnancy**	**13,262**				
No documentation of ART in pregnancy	489	1.70 (1.39, 2.08)	< 0.001	1.37 (1.03, 1.82)	0.032
ART initiated prior to pregnancy	8598	Ref	Ref	Ref	Ref
ART initiated during pregnancy	4175	0.86 (0.80, 0.93)	< 0.001	0.90 (0.81, 1.00)	0.048
**Gravida**	**13,323**				
Primigravida	1953	0.85 (0.77, 0.94)	0.001	0.88 (0.77, 1.00)	0.057
Multigravida	11,370	Ref	Ref	Ref	Ref

*OR* Odds ratio (from univariable analysis), *aOR* Adjusted odds ratio (from multivariable analysis)

### Viral load availability

HIV RNA availability was among the strongest predictors for breastfeeding in the multivariable analysis. Among the 13,354 women included, 11,161 had data on viral load documentation status, of which only 2865 (25.7%) had a documented HIV RNA result available at delivery. Of these, 2711 (94.6%) were virally suppressed (< 400 copies/mL). Women who had a documented viral load during pregnancy were more likely to breastfeed compared with women that did not have a documented viral load (adjusted odds ratio (aOR) 0.59; 95% CI 0.54, 0.65; *P*-value < 0.001). Among women with a documented viral load, 50.8% (*n* = 1456) chose to breastfeed at discharge while only 35.4% (*n* = 2936) of women without a documented viral load during pregnancy chose to breastfeed.

There were only 153 (5.4%) women identified with HIV RNA > 400 copies/mL in pregnancy, and of these, 46 (30.1%) chose to breastfeed (contrary to Botswana guidelines). Likewise, breastfeeding was observed among 134 (27.4%) of 489 women with no reported ART use in pregnancy (also contrary to the Botswana guidelines). We did note an increase in HIV RNA availability in the obstetric record over time, with 38.5% of participants having a documented viral load in 2018/2019 compared with 9.5% in 2016/2017, representing a favourable trend.

### Electronic laboratory record verification

We separately evaluated electronic national laboratory records for 150 women who had documentation of HIV RNA in pregnancy and 150 women who did not. Of these, 140 (93.3%) with a documented HIV RNA result on the obstetric record had confirmation of that test in the laboratory database. For women with no documented HIV RNA result in the obstetric record, 93 (62.0%) women had an HIV RNA test conducted during pregnancy, according to the laboratory database. This finding indicates that the lack of available viral load results at the time of discharge from the maternity ward was likely a combination of incomplete testing in pregnancy and incomplete recording of test results onto the obstetric record.

## Discussion

We evaluated maternal feeding choice among WLHIV and identified ongoing low levels of breastfeeding following the 2016 guideline change in Botswana, and evidence that the requirement for a suppressed HIV RNA result during pregnancy may be a barrier to breastfeeding. To our knowledge, this is the first published data on feeding choices in Botswana after the new guidelines were implemented and in the ‘treat all’ ART era.

Breastfeeding among WLHIV in Botswana was low overall despite the guideline shift in 2016, with only 39.7% of women breastfeeding upon discharge from the maternity ward and an increase in breastfeeding of only 1.3% from 2016/2017 to 2018/2019. While this level of breastfeeding is higher than previous reports from Botswana before 2016 where fewer than 20% of WLHIV chose to breastfeed [14, 15], it is significantly lower than in other African settings where studies have reported choice to exclusively breastfeed by 80–90% of WLHIV [16, 17]. Most African countries with the highest HIV prevalence have adopted a public health approach rather than an individualized approach to support breastfeeding for WLHIV [18, 19]. In 2011, Botswana’s neighbouring country, South Africa, committed to promoting exclusive breastfeeding for WLHIV following the WHO 2010 guidelines on HIV and infant feeding and ended the universal formula programme for WLHIV. In contrast, Botswana continues to provide infant formula free-of-charge for all WLHIV who choose to formula feed.

Our hypothesis that the requirement for documented HIV RNA suppression in pregnancy served as a barrier to breastfeeding was confirmed. Only 25.7% of women had an HIV RNA (viral load) result documented in the obstetric record overall, and the presence of a result (95% of which were virally suppressed) did correlate with the choice to breastfeed. This low availability was only partially reflective of actual HIV RNA testing in pregnancy, as our electronic record verification identified that 62% of women with no documented HIV RNA result on the obstetric record did have an HIV RNA test performed during pregnancy. Thus, one implication of our findings is that improved documentation of HIV RNA results in the obstetric record (or improved access of maternity nurses to the electronic records) could improve breastfeeding uptake. However, we also noted an increase in available HIV RNA results over time that was larger than the increase in breastfeeding, suggesting that other factors may have also played a role in the reluctance to choose to breastfeed. Feeding decisions may have been influenced by earlier guidance in Botswana which emphasized formula feeding, fear of MTCT [20], ambiguous/confusing messages regarding infant feeding [20, 21] and lack of social supports for breastfeeding among WLHIV [22]. In addition, while most HIV RNA results were undetectable (< 400 copies/mL), some women without a result or with unsuppressed HIV RNA still chose to breastfeed, indicating that factors beyond MTCT risk were likely involved in feeding decisions.

We identified additional factors associated with lack of breastfeeding in our multivariable analysis, most of which were consistent with prior studies [20, 23]. Lack of prior antenatal visits was a strong risk factor, which may have been related to lack of a possible HIV RNA result in pregnancy or differences in counselling practices for these women. Women who delivered in a tertiary hospital were less likely to breastfeed compared with women who delivered in a primary/district hospital, but it is not known whether this was related to differences in patient populations at these tertiary centres, counselling differences, or other factors associated with urban locations. We also found age, education, nationality, and ART status during pregnancy to be associated with feeding choice.

Our study had several limitations. We were only able to evaluate feeding method at the time of maternity ward discharge. The decision to breastfeed at the time of discharge from maternity does not equate with exclusive breastfeeding for the WHO recommended 6 months. A prospective study would be needed to examine breastfeeding patterns throughout infancy. While there is a high percentage of viral suppression in Botswana overall (85%) [6], the 95% suppression in this report may be artificially high if women who tested were those most motivated by PMTCT concerns. However, multiple prior studies in Botswana support this high level of suppression in pregnancy, and we believe this indicates a true low MTCT risk for most breastfeeding women [5, 14]. Strategies that evaluate time on ART and adherence to ART that can be performed by maternity nurses throughout ANC and at the time when infant feeding decisions occur may be a useful alternative to reliance on HIV RNA documentation.

## Conclusions

Despite a guideline shift in 2016 designed to support breastfeeding among WLHIV, fewer than 40% of WLHIV in Botswana from 2016 to 2019 chose to breastfeed their infants. This low rate of breastfeeding underscores the need to address barriers affecting feeding choices, including the requirement for documented HIV RNA suppression. While additional barriers to breastfeeding exist and should be explored, in a setting of long-standing use of suppressive ART with almost two-thirds of WLHIV conceiving on ART and high overall suppression, the requirement for documented HIV RNA suppression is unlikely to prevent a substantial amount of HIV transmission during breastfeeding but likely contributes to low rates of breastfeeding.

## Data Availability

Data are not publicly available but may be provided by the corresponding and senior authors upon reasonable request and with permission of the Institutional Review Boards which oversee the study.

## Abbreviations

ANC: Antenatal clinic
aOR: Adjusted odds ratio
ART: Antiretroviral therapy
CI: Confidence interval
HIV: Human Immunodeficiency Virus
IDCC: Infectious disease control clinic
OR: Odds ratio
PMTCT: Prevention of mother-to-child transmission
RNA: Ribonucleic acid
WHO: World Health Organization
WLHIV: Women living with HIV
